# Experimental and Theoretical Investigation on Heat Transfer Enhancement in Micro Scale Using Helical Connectors

**DOI:** 10.3390/ma17051067

**Published:** 2024-02-26

**Authors:** Malyne Abraham, Zachary Abboud, Gabriel Herrera Arriaga, Kendall Tom, Samuel Austin, Saeid Vafaei

**Affiliations:** Mechanical Engineering Department, Bradley University, Peoria, IL 61625, USA

**Keywords:** nanofluid, heat transfer coefficient, microchannel, helical flow, helical connector

## Abstract

Microscale electronics have become increasingly more powerful, requiring more efficient cooling systems to manage the higher thermal loads. To meet this need, current research has been focused on overcoming the inefficiencies present in typical thermal management systems due to low Reynolds numbers within microchannels and poor physical properties of the working fluids. For the first time, this research investigated the effects of a connector with helical geometry on the heat transfer coefficient at low Reynolds numbers. The introduction of a helical connector at the inlet of a microchannel has been experimentally tested and results have shown that this approach to flow augmentation has a great potential to increase the heat transfer capabilities of the working fluid, even at low Reynolds numbers. In general, a helical connector can act as a stabilizer or a mixer, based on the characteristics of the connector for the given conditions. When the helical connector acts as a mixer, secondary flows develop that increase the random motion of molecules and possible nanoparticles, leading to an enhancement in the heat transfer coefficient in the microchannel. Otherwise, the heat transfer coefficient decreases. It is widely known that introducing nanoparticles into the working fluids has the potential to increase the thermal conductivity of the base fluid, positively impacting the heat transfer coefficient; however, viscosity also tends to increase, reducing the random motion of molecules and ultimately reducing the heat transfer capabilities of the working fluid. Therefore, optimizing the effects of nanoparticles characteristics while reducing viscous effects is essential. In this study, deionized water and deionized water–diamond nanofluid at 0.1 wt% were tested in a two-microchannel system fitted with a helical connector in between. It was found that the helical connector can make a great heat transfer coefficient enhancement in low Reynolds numbers when characteristics of geometry are optimized for given conditions.

## 1. Introduction

In the evolving landscape of thermal management, innovative techniques are being studied to enhance the heat transfer capabilities of cooling management systems in an attempt to improve energy and economic efficiency. Among these, passive heat transfer enhancement methods are gaining traction for their ability to improve system performance without the need for external power sources. These methods involve strategic modifications to the cooling system’s internal flow patterns or the working fluid itself. For example, they incorporate specific geometries or inserts into the system that alter the flow path to induce turbulence or secondary flow patterns [1]. These alterations in flow dynamics can significantly enhance the heat transfer rate without the need for additional external energy. For instance, the implementation of helical connectors or other specially designed geometrical structures in the flow path can disrupt the laminar flow and promote the random motion of molecules and possible nanoparticles, thereby increasing the heat transfer coefficient and improving the efficiency of the cooling system. When combined with the superior thermal conductivity of nanofluids, these two passive techniques can synergistically enhance the overall heat transfer performance, offering a promising approach for advanced thermal management solutions. The advent and application of nanofluids have marked a significant advancement in the field. Defined as fluids containing suspended nanoparticles, nanofluids have demonstrated potential in various fields ranging from automotive to electronic cooling systems. Their allure lies in the promise of enhanced thermal properties compared to traditional working fluids.

Choi and Eastman [2] revolutionized thermal science when it was discovered that dispersing nanoparticles into base fluids like water, oil, or ethylene glycol significantly enhances thermal conductivity. This enhancement is pivotal because the thermal conductivity of a working fluid is a critical factor in improving heat transfer efficiency. However, the improvement in thermal conductivity often comes with the trade-off of increased viscosity [3,4]. The increased viscosity can impede fluid flow, negatively impacting the overall heat transfer process. Characteristics of nanoparticles, including concentration and temperature, can also have a significant impact on the heat transfer capabilities of a nanofluid [4,5,6,7,8]. The combination of these parameters is specifically important in microscale applications. Several studies have utilized nanofluids in micro- and minichannels to investigate how they increase the heat transfer capabilities of the working fluid. For example, Gupta and Subbarao [9] employed the use of Al_2_O_3_–deionized water nanofluids at mass concentrations of 1–4% in an empirical study of a microchannel heat sink at Reynold numbers ranging from 10 to 50. They found that by simply adding nanoparticles into the base fluid, the heat transfer coefficient increased by 43.34% when compared to that of the base fluid with a Reynolds number of 50 and a particle concentration of 4%. An increase in heat transfer enhancement was seen to be a function of both increased Re and increased nanoparticle concentration. They suggested this enhancement may be due to increased thermal conductivity, surface area, and increased particle collisions due to the nanoparticles present in the base liquid. However, the addition of nanoparticles also increased the friction factor and pressure drop due to higher viscosities associated with the nanofluids. Ho et al. [10] studied how alumina nanoparticles in ultrapure water at volume concentrations of 0.5% and 1.0% affected the thermal performance of a microchannel heat sink. They found that the addition of nanoparticles resulted in a higher heat transfer rate when compared to that of the base fluid. They suggested this may be due to enhanced thermal conductivity and a reduction in the thermal boundary layer as nanoparticles moved towards the channel walls. Additionally, as nanoparticle concentration increased, these effects became more pronounced. Furthermore, an increase in Reynolds number from 500–1500 resulted in convective heat transfer enhancement for all cases, with greater enhancement seen at the higher particle concentration, possibly due to the higher thermal conductivity associated with this mixture and increased particle collisions. Liu et al. [11] numerically investigated the use of hybrid carbon nanotubes (CNT)/Fe_3_O_4_/water nanofluids in a minichannel hairpin heat exchanger at Reynolds numbers ranging from 500–2000. Their results indicated that as nanoparticle concentration is increased, average heat transfer coefficient within the minichannel increased. Additionally, an increase in inlet water temperature resulted in the simultaneous effect of increased heat transfer coefficient and decreased fluid viscosity. They suggested this may be due to the temperature dependence of thermal conductivity and viscosity of the nanofluids, and, as temperature increases, thermal conductivity tends to increase as viscosity tends to decrease. They also noted that as inlet temperature increased, fluid velocity at the wall and central tube region increased and decreased, respectively, possibly due to the decrease in viscosity near the wall, a result of increased temperature in this region. These studies highlight the complex interplay of particle concentration, temperature, and flow characteristics on the heat transfer performance of microscale thermal management systems.

Nanodiamonds (NDs), specifically, especially when dispersed in various base fluids such as ethylene glycol/water mixtures, have demonstrated significant enhancements in thermal properties. These nanoscale particles, typically about 10 nm in size, notably improve the thermal conductivity of the fluids in which they are dispersed; however, viscosity also tends to increase. For example, Mashali et al. [12] showed that a 1.0% concentration of ND in various ethylene glycol/water base fluids led to an increase in thermal conductivity of up to 17.8% in the base fluid with a ratio of 20:80 ethylene glycol/water at 60 °C. The inherent high thermal conductivity of diamond, the base material for nanodiamonds, significantly contributes to these enhancements. Diamond’s thermal conductivity is exceptionally high, typically exceeding 2200 W/m∙K, and can reach up to 3320 W/m∙K in isotropically pure monocrystalline synthetic diamond. This remarkable thermal conductivity is attributed to the strong carbon–carbon covalent bonds and the low photon scattering in diamond’s structure [12]. The viscosity of nanodiamond fluids is also a critical aspect, directly impacting their applicability in thermal systems. Adding nanodiamonds to a base fluid can lead to an increase in viscosity, which is generally undesirable due to the potential for energy loss [12]. This increase in viscosity can be influenced by factors such as nanodiamond concentration and particle size. Alshayji et al.’s [13] research into the thermal properties of water-based diamond nanofluids displayed this trend when their results showed that the viscosity of diamond nanofluids rises with particle loading and decreases with temperature. The most pronounced increase in viscosity observed in their study was over 36% at 20 °C and a volume concentration of 1.25%, the lowest temperature and highest concentration tested. Conversely, a minimal increase in viscosity of less than 3% was seen at the lowest concentration and highest temperature tested, 0.125% and 60 °C, respectively. This finding is critical as it reflects the typical behavior of nanofluids, where viscosity changes in response to both temperature and particle concentration.

Inherent thermal properties of nanofluids are also significantly influenced by dispersion stability. Physically unstable nanofluids, characterized by dispersion instability (nanoparticle aggregation within the fluid) and kinetic instability (sedimentation of agglomerated particles due to gravity), significantly affect the functional efficacy of nanofluids [14,15]. The development of stable nanofluids has continued to be a challenge, especially in the case of nanodiamond nanofluids, possibly due to the high number of carbon impurities present in the ultra-dispersed diamond (UDD) nanoparticles prepared by the detonation method [16]. Due to their tendency to agglomerate, several methods have been employed for further purifying the diamond nanoparticles and creating higher dispersion stability. These methods include purification via the use of strong acids and modification by surface functionalization [12,17]. For example, Sundar et al. [16] compared water-based UDD diamond nanofluid with a water-based carboxylated nanodiamond (ND) nanofluid, where the UDD powder was treated with sulfuric acid to remove the carbon impurities. Their results showed that the carboxylated ND resulted in a smaller average particle size and higher dispersion stability, especially for cases where pH was above 8, and demonstrated superior thermal performance due to the formation of carboxyl groups on the surface of the ND and the greater fluid stability. It was also suggested that ultrasonication can further facilitate the nanofluids’ stability by breaking up agglomerated particles into single nanodiamond particles homogenously dispersed within the fluid, allowing for higher particle concentrations to be utilized and maximum thermal conductivities to be reached. Dispersion stability is particularly important in micro-scale applications, where particle agglomeration and sedimentation can cause blockage and significant damage to the system. Additionally, uniformity throughout the nanofluid helps facilitate consistent thermophysical properties and efficient and accurate heat transfer.

While altering the fluid characteristics directly with the use of nanofluids is becoming a promising area of study, additional passive techniques for heat transfer enhancement are also being investigated. One such method is through alteration of the geometry of the fluid channel. Several different geometric configurations have been investigated for their impacts on fluid flow augmentation and their potential to increase the heat transfer capabilities of the system. For example, Ebrahimi et al. [18] numerically investigated how the flow patterns in mini twisted oval tubes could enhance the heat transfer coefficient of water. In this study, they altered the geometry of the tube by changing the cross-sectional aspect ratio (defined as the major axis diameter over the minor axis diameter) while maintaining a constant hydraulic diameter of 5 mm. Additionally, the Reynolds number was varied from 500–1100. Their results showed that the twisted oval tubes had higher heat transfer performance than a traditional circular tube for all geometries studied. They suggested that the heat transfer enhancement may be due to the secondary flow patterns created by the twisted wall geometry. The secondary spiral flow patterns may alter the thickness of the thermal boundary layer while simultaneously intensifying turbulent flow and fluid mixing, ultimately increasing the heat transfer coefficient. Furthermore, as Re and aspect ratio increased, further heat transfer enhancement was seen, which was suggested to be a result of convection promotion. Ebrahimi et al. [19] also numerically studied conjugated heat transfer enhancement in a rectangular microchannel heat sink by altering the channel geometry with the addition of longitudinal vortex generators. In this study, Al_2_O_3_/water and CuO/water nanofluids at particle volume fractions of 0.5–3.0% were used as the working fluid, and the Reynolds number was varied between 100–1000. Their results showed that as the Reynolds number increased, the recirculation zones behind the longitudinal vortex generators became more pronounced. They suggested that this effect may promote better fluid mixing and result in thermal boundary layer reduction, ultimately leading to an enhancement in the Nusselt number. Additionally, the use of nanofluids resulted in better heat absorption of the working fluid and a further enhancement in the Nusselt number, showing enhancements of up to 30.63% and 53.06% for Al_2_O_3_ and CuO nanofluids, respectively. They suggested the addition of nanoparticles provided a positive impact due to their greater thermal conductivities while simultaneously increasing the available heat transfer surface area of the fluid and molecular random motion, ultimately leading to superior heat transfer capabilities when compared to that of water.

Geometry variation to augment fluid flow patterns and improve heat transfer was also studied by Cheng et al. [20]; they numerically investigated the effects of combining a triangle protrusion, a corrugated surface, and an oblique angled upper plate in a heat sink using Al_2_O_3_–water at various concentrations as the working fluid. In this study, flow parameters, such as the Reynolds number, and structural parameters, such as the height of the triangular protrusion, the amplitude, wavelength and wavelength ratio of the corrugated surface, and the angle of the upper plate, were varied to determine their effects on the overall heat transfer performance of the heat sink. Their results showed that simultaneously altering the geometry of the heat sink and utilizing nanoparticles within the base fluid can enhance both the heat transfer capabilities and the temperature uniformity within the heat sink. They suggested this may be due to increases in the fluid velocity and reduction of the low velocity regions from the triangular protrusion; improved jet velocity and reduced fluid boundary layers from the corrugated surface; and increased fluid velocity due to the angle of the upper plate. An optimal structure and concentration were numerically found, resulting in a 26% increase and 67% decrease in the average Nusselt number and the temperature standard deviation, respectively. In a similar study by Naranjani et al. [21], the thermal performance of adding Al_2_O_3_–water nanofluids at volume fractions of less than 4% in a corrugated channel heat sink was compared to that of water and a heat sink with straight channels. Their results showed that altering the geometry of the heat sink with corrugated channels, as opposed to straight channels, and utilizing Al_2_O_3_–water nanofluids enhanced the heat transfer performance by 22–40%, with the tradeoff of increased pumping power requirements. They suggested that the corrugated channels may induce a secondary flow pattern, recirculating the hot fluid from the channel wall to the central region and back to the channel wall. This effect is enhanced by increasing the Reynolds number and the curvature of the channel, which further alters the fluid flow patterns and heat transfer surface area, ultimately leading to increased heat transfer capabilities.

The unique geometry of helical tubes can also significantly alter fluid flow characteristics compared to straight tubes, primarily due to the effects of centrifugal forces and the development of secondary flow fields [22,23]. For example, Zonouzi et al. [23] performed a 3D numerical study on the effects of laminar nanofluid flow through a helical tube. The effects of helical diameter, pitch, and nanofluid concentration were all investigated for their impact on the thermal behavior of the nanofluid. Their results showed that helical diameter plays a key role in the centrifugal force acting on the fluid within the pipe. This force induces a secondary flow along the cross-section of the pipe, causing the fluid within the core of the tube to move towards the outer bend, creating a maximum velocity zone parallel to the pipe wall and a temperature drop in this region. This secondary flow directly impacts the heat transfer rate of the fluid. With the addition of nanoparticles into the base fluid, the overall heat transfer coefficient was further enhanced, due to the improved thermal conductivity of the base fluid, a result of the nanoparticles present within the fluid. Additionally, the nanofluid had a much greater effect on enhancing the overall heat transfer coefficient in the helical tubes when compared to straight tubes. The pitch was also altered to determine the effects it has on the heat transfer capabilities of the working fluid. It was found that the effects of pitch are much less prominent than the effects of other parameters, such as helical diameter. However, as pitch increased, the overall heat transfer coefficient generally increased. They suggested that the pitch induces torsion on the fluid flow, and as pitch increases, this torsion causes a downward shift of the high velocity region [23]. Furthermore, the Reynolds number in helical tubes is vital for determining the flow regime (laminar or turbulent) and thereby influences both the heat transfer and pressure drop characteristics. In helical tubes, the onset of turbulence is delayed compared to straight tubes due to the presence of secondary flow caused by the curvature. This secondary flow may act as a stabilizer on the laminar flow regime within the tube [24]. This phenomenon impacts the critical Reynolds number for turbulence, making it a key factor in designing and operating systems using helical tubes. Overall, tube geometry variations can have a significant impact on the heat transfer capabilities of a microscale thermal management system. When these effects are combined with the superior thermal properties of nanofluids, optimal cooling systems can be engineered to meet specific heat transfer requirements.

The objective of this study was to investigate the enhancement of heat transfer coefficients at micro and nano scales under low Reynolds numbers. Passive methods were examined to boost the heat transfer efficiency in microchannels by altering flow characteristics and increasing the random motion of molecules and potential nanoparticles. This was achieved through the integration of a helical connector at the microchannel inlet, with a focus on how its geometric features influenced flow dynamics and molecular movement. Additionally, a diamond nanofluid was employed to assess whether its superior thermal properties could further improve heat transfer compared to conventional base liquids like deionized water. The findings of this study reveal heat transfer coefficient enhancement following the fluid’s passage through the helical connector, underscoring the connector’s effectiveness in enhancing molecular and nanoparticle motion. Notably, the helical connector showed a greater potential to improve heat transfer coefficients than merely adding nanoparticles to base liquids. The results were also compared with those from a previous study by the same authors [25] using a nozzle-shaped connector, finding that the helical design significantly outperforms the nozzle in enhancing the working fluid’s heat transfer capabilities under similar conditions.

## 2. Materials and Methods

### 2.1. Experimental Setup

**Figure 1:**
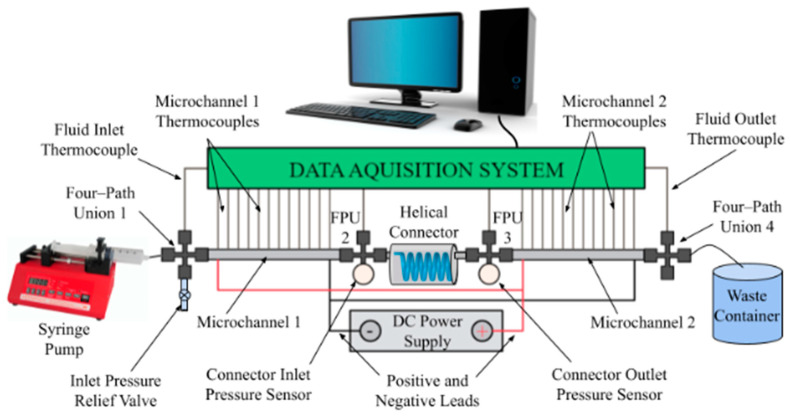
Schematic of two-microchannel experimental setup with connector in between.

**Figure 2:**
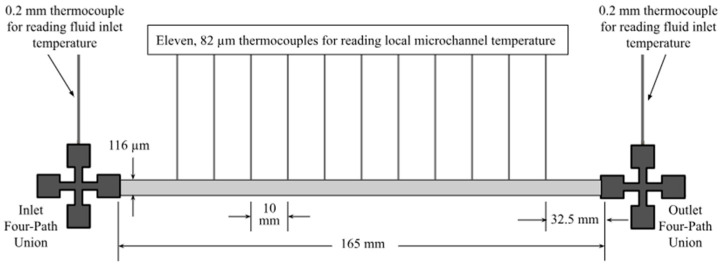
Enlarged, dimensioned schematic of a stainless-steel microchannel showing how temperature distribution measurements were taken at the inlet and outlet and along the channel.

**Figure 3:**
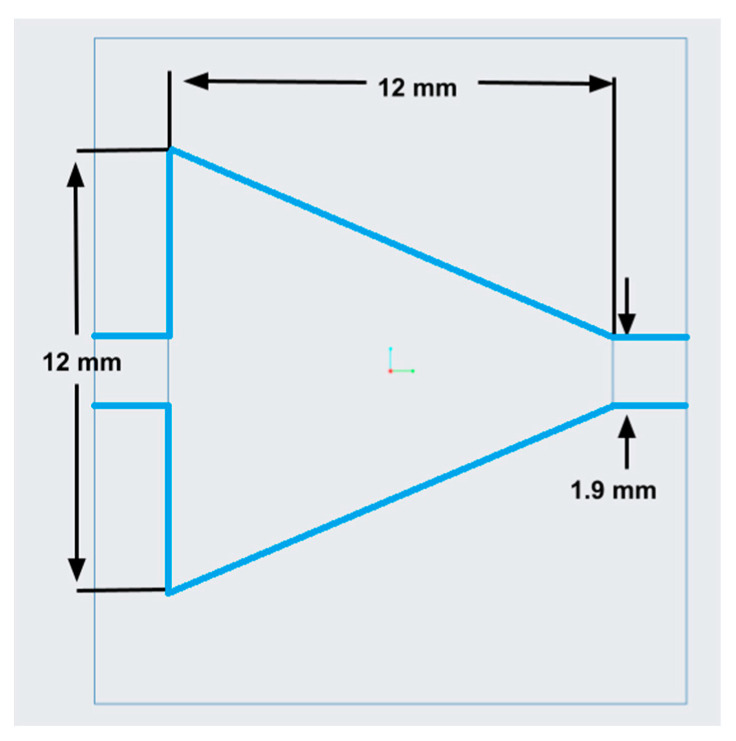
Dimensioned diagram of the nozzle-shaped connector used for result comparison.

A two-microchannel system was designed to identify how the use of a connector may affect and enhance the heat transfer capabilities of the working fluid. The schematic of the experimental setup is shown in Figure 1. An enlarged, dimensioned schematic of a microchannel displaying temperature measurement locations is shown in Figure 2. Multiple helical connectors were implemented to study how a change in pitch, length, and/or helical diameter affected the fluid flow and, therefore, altered the heat transfer coefficient. Results were compared to a nozzle-shaped connector; the geometry of the nozzle connector is shown in Figure 3 and can be found in reference [25].

A New Era Systems’ Ne-8000 pump (Farmingdale, NY, USA) equipped with a Chemglass 50 mL syringe was used to introduce the working fluid into the system, where it then flowed into a segment of Luer-Lock tubing (Hamilton 86510, De Pere, WI, USA). Four-path unions (FPU) (Cole-Parmer EW-02008-26 High-Pressure Union, Vernon Hills, IL, USA) were placed at the inlet and outlet of each microchannel, and all were fitted with a 0.2 mm K-type thermocouple (Omega Engineering 5TC-TT-K-30-36, Norwalk, CT, USA) to read the fluid temperature at the inlet and outlet of each microchannel. The first four-path union was also equipped with an in-line pressure relief valve (Industrial Specialties Mfg. PRV-30#-PC, Englewood, CO, USA) rated at a cracking pressure of 30 psi, allowing fluid to escape if pressure drop reached potentially damaging levels. Fluid flowed through the first four-path union into the first stainless-steel microchannel. Each stainless-steel microchannel had an internal diameter of 116 μm and a length of 165 mm (Hamilton 23s gauge, De Pere, WI, USA) and was equipped with 11 82 μm thermocouples (RS Pro 397-1589 K-type, Auckland, New Zealand) placed 10 mm apart, attached with thermally conductive epoxy (Duralco 132 Resin and Hardner, Brooklyn, NY, USA), to read the temperature distribution along the stainless-steel channel. For thermal insulation, a 1 cm layer of 3M scotch-weld 2214 epoxy glue (2214-HD-GRAY-6OZ, 3M, St. Paul, MN, USA) was added, surrounding each microchannel to ensure heat was not lost to the surroundings and accurate measurements were taken.

Upon leaving the first microchannel, the fluid entered the second four-path union. Both the second and third four-path unions were fitted with pressure transducers (Omega Engineering PX26-005GV, Norwalk, CT, USA) powered by their external power supply (Omega Engineering PST 4130, Norwalk, CT, USA) to measure the connector inlet and outlet pressures, respectively. This data was used to determine pressure drop through the helical connector. Fluid flowed from the second four-path union into the helical connector. Helical connectors were modeled using Creo Parametric and then 3D printed on a Form 3+ resin type printer from Formlabs. Upon leaving the connector, the fluid flowed through the third four-path union into the second stainless-steel microchannel, exiting through the last four-path union into a waste container.

Each stainless-steel microchannel was resistively heated by attaching positive and negative leads near the inlet and outlet of each microchannel. The leads were connected to a DC power supply (Sorenson XPH 20-20, Ameteck, Berwyn, PA, USA), and the current was manually adjusted until a maximum temperature of 85 °C was read from the thermocouples. All measurements were taken at steady-state, and care was taken to ensure that the temperature at any point within the system did not exceed 90 °C to avoid boiling of the working fluid.

All data was acquired using an NI cDAQ-9178 Data Acquisition System (National Instruments, Austin, TX, USA). The NI-9213 module was utilized to obtain the temperature readings from the thermocouples, while a NI-9218 module coupled with a NI-9982 adapter was used to collect the pressure readings across the connector. LabVIEW programming software (National Instruments, 2020 sp1) was employed to collect and record all measurements.

### 2.2. Nanofluid Synthesis

For this experiment, diamond nanofluid was purchased from US Research Nanomaterials, Inc. (Houston, TX, USA). The fluid contained diamond nanoparticles with a spherical morphology at a purity of 98.3% with an average particle size of 3 nm, made by the explosion synthesized method. The diamond nanofluid was obtained fully dispersed in water at an initial concentration of 5 wt% and then diluted with deionized water to reach a final concentration of 0.1 wt%. After dilution, the nanofluid was magnetically stirred for 45 min to ensure good mixing of the pre-dispersed fluid with the deionized water, producing a homogeneous, stable nanofluid.

To determine heat transfer coefficient in future calculations, physical properties of the diamond nanofluid were first defined. Calculation of density and specific heat required that concentration of the nanofluid be converted from weight percent to volume percent by the following equation [26]:

(1) $$\phi =\frac{w\rho_{f}}{\rho_{ND}\left(1-w\right)+w\rho_{bf}}\;,$$

where ϕ is the concentration in volumetric percent, w is the weight percent, and $\rho_{ND}$ and $\rho_{bf}$ are the density of the diamond nanoparticles and base fluid (deionized water), respectively. Density of the diamond nanofluid could then be obtained by the following equation [26]:

(2) $$\rho_{nf}=\left(1-\phi \right)\rho_{bf}+\phi \rho_{ND},$$

where $\rho_{nf}$ is the density of the diamond nanofluid. Specific heat of the diamond nanofluid was also necessary to determine for future calculations and was determined by [20]:

(3) $$c_{p,nf}=\frac{\phi {\left(\rho c_{p}\right)}_{ND}+\left(1-\phi \right){\left(\rho c_{p}\right)}_{bf}}{\rho_{nf}},$$

where $c_{p}$ is the specific heat, and the subscripts nf, ND, and bf represent the diamond nanofluid, diamond nanoparticles, and base fluid, respectively.

All experiments were conducted so that the average temperature throughout the system was approximately 50 °C. The thermal conductivity for water and diamond nanofluid at 0.1 wt% were obtained from research, and the values are tabulated in Table 1.

### 2.3. Determining Heat Transfer Coefficient

Analyzing and determining the heat transfer coefficient for nanofluid-based cooling systems requires a fundamental understanding of the system parameters, such as mass and volume flow rates, fluid temperature profiles along the channels, and heat flux through the system. In this study, local heat transfer coefficient was investigated as a function of non-dimensional location at various Reynolds numbers for diamond nanofluid and compared to that of water; thus, each chosen Reynolds number dictated the necessary volumetric flow rate of the fluid through the system, which was determined by the following equation:

(4) $$\dot{V}=\frac{Re\mu A}{\rho D},$$

where $\dot{V}$ is the volumetric flow rate, Re is the chosen Reynolds number, μ is the dynamic viscosity of the fluid, A is the cross-sectional area of the microchannel, ρ is the density of the fluid, and D is the internal diameter of the microchannel. It was necessary to convert volumetric flow rate to mass flow rate in order to define other foundational parameters required for calculating heat transfer coefficient by

(5) $$\dot{m}=\dot{V}\rho ,$$

where $\dot{m}$ is the mass flow rate. Heat flux through each microchannel could then be determined by the following equation:

(6) $${\dot{q}}^{\prime }=\frac{\dot{m}c_{p}(T_{out}-T_{in})}{\pi Dl},$$

where ${\dot{q}}^{\prime }$ is the heat flux, $c_{p}$ is the specific heat capacity of the fluid, $T_{out}$ and $T_{in}$ are the measured temperatures at the outlet and inlet of the microchannel, respectively, and $D_{c}$ and $l_{c}$ are the internal diameter and the length of the microchannel, respectively. By determining heat flux, it is then possible to calculate the instantaneous temperature at any point along the microchannel by use of the following equation:

(7) $$T_{f}\left(x\right)=T_{in}+\frac{{\dot{q}}^{\prime }\pi D}{c_{p}\dot{m}}x,$$

where $T_{f}\left(x\right)$ is the local fluid temperature calculated a distance x from the inlet of each microchannel. With all the necessary variables defined, local heat transfer coefficient was determined at each location along the microchannel by the following equation:

(8) $$h\left(x\right)=\frac{{\dot{q}}^{\prime }}{T_{s}\left(x\right)-T_{f}\left(x\right)},$$

where h(x) is the local heat transfer coefficient calculated a distance x from the inlet of the microchannel, and $T_{s}\left(x\right)$ and $T_{f}\left(x\right)$ are the microchannel surface and fluid temperatures, respectively, measured a distance x from the inlet of the channel. The local heat transfer coefficient quantifies the efficiency of the working fluid’s ability to transfer heat from the microchannel surface to the fluid, which is the focus of this study.

## 3. Results and Discussion

**Figure 4:**
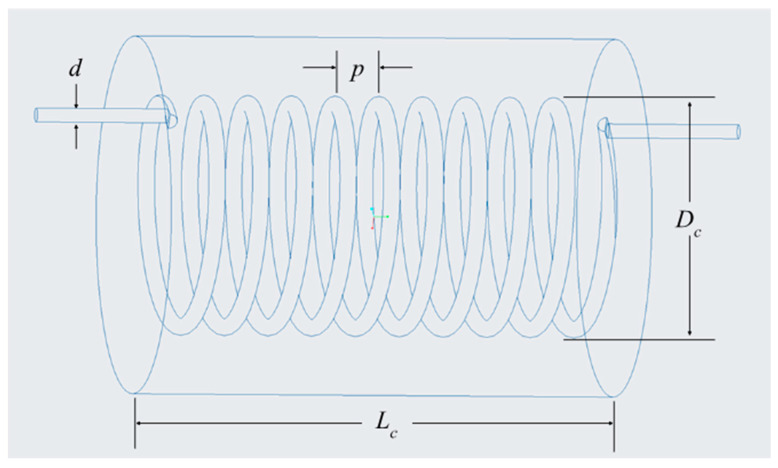
Schematic of a helical connector with labeled geometric parameters.

This study aims to enhance the random motion of molecules and potential nanoparticles within a fluid by inducing a secondary flow. This is achieved by incorporating a helical connector immediately upstream of a microchannel’s inlet. The secondary flow, which manifests as a cross-sectional circulatory motion, is generated when the fluid moves through the helical structure. This phenomenon is driven by the curvature of the channel, which imposes a centrifugal force on the fluid [18,22,28]. Additionally, it has been noted that such secondary motion can serve as a stabilizing factor, effectively elevating the critical Reynolds number for the flow [24,28]. Figure 4, below, is a schematic showing the interior design features of a helical connector, where $D_{c}$ represents the helical diameter, d denotes the inner diameter of the channel in which the fluid flows, p is the pitch of the helix, and $L_{c}$ signifies the length of the connector. The geometry of helical connectors will be modified for future works to have more stable and consistent data in both channels. Table 2, below, shows the specific dimensions used for the helical connectors used in this study. The interior geometry of each connector was altered based on these four parameters, and the specific dimensions used are also labeled on the corresponding CFD figures and graphs below for clarity.

Generally, the role of the helical connector may vary; it may serve as a mixer or a stabilizer, based on the specific conditions. As a mixer, the helical connector enhances the random motion of molecules and potential nanoparticles, but beyond certain conditions, it might transition to acting as a stabilizer. In the stabilizing phase, the molecular (and particle) random motion is dampened. With a decrease in the connector’s diameter, under set conditions, the centrifugal force intensifies, potentially amplifying the secondary motion up to a point where the connector begins to stabilize the flow, leading to a reduction in the heat transfer coefficient. Likewise, if the length of the connector is extended, the secondary motion may increasingly serve a stabilizing function. Consequently, the heat transfer coefficient may diminish subsequent to the helical connector’s stabilizing mode.

For nanofluids, the secondary flow can promote uniform distribution within the mixture, while the centrifugal force might segregate nanoparticles from the base fluid. When the secondary flow functions as a mixer or homogenizer, it can enhance the nanofluids’ heat transfer capabilities. Moreover, this cross-sectional circulatory motion can significantly improve energy exchange across the fluid layers in laminar flow conditions, thereby boosting the heat transfer coefficient when it operates as an effective mixing mechanism.

**Figure 5:**
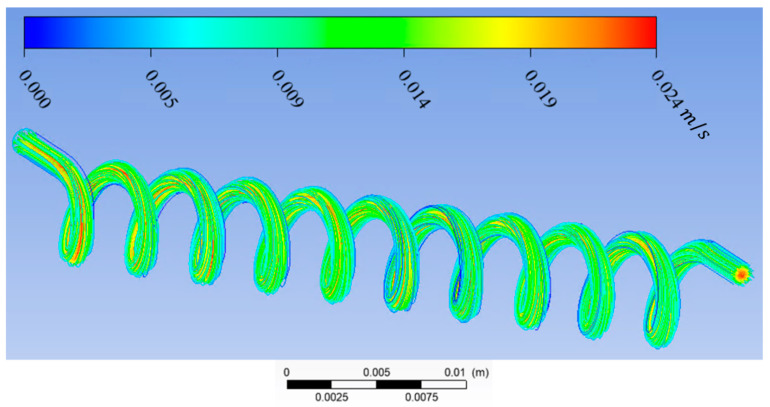
Streamlines in a helical connector when $L_{c}=40\;\mathrm{mm}$, $D_{c}=5\;\mathrm{mm}$, d=1.5 mm, p=4 mm. Working fluid was deionized water, and Re = 400.

**Figure 6:**
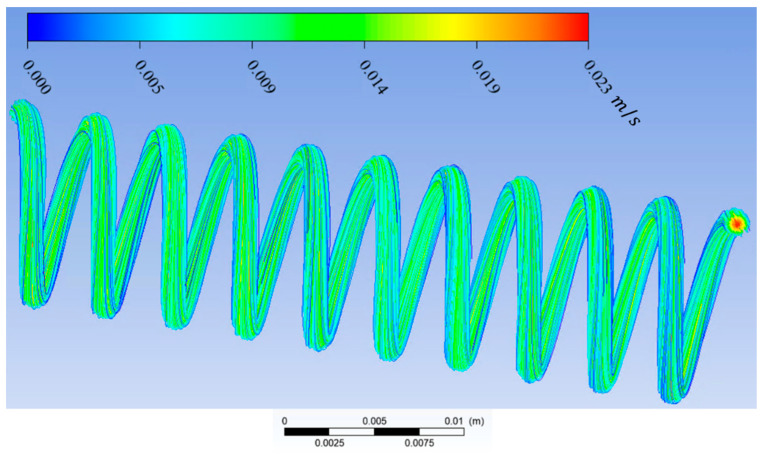
Streamlines in a helical connector when $L_{c}=40\;\mathrm{mm}$, $D_{c}=10\;\mathrm{mm}$, d=1.5 mm, and p=4 mm. Working fluid was deionized water, and Re = 400.

**Figure 7:**
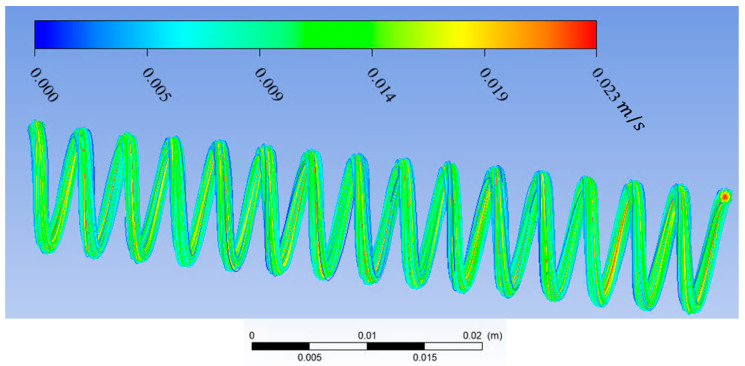
Streamlines in a helical connector when $L_{c}=60\;\mathrm{mm}$, $D_{c}=10\;\mathrm{mm}$, d=1.5 mm, and *p* = 4 mm. Working fluid was deionized water, and Re = 400.

**Figure 8:**
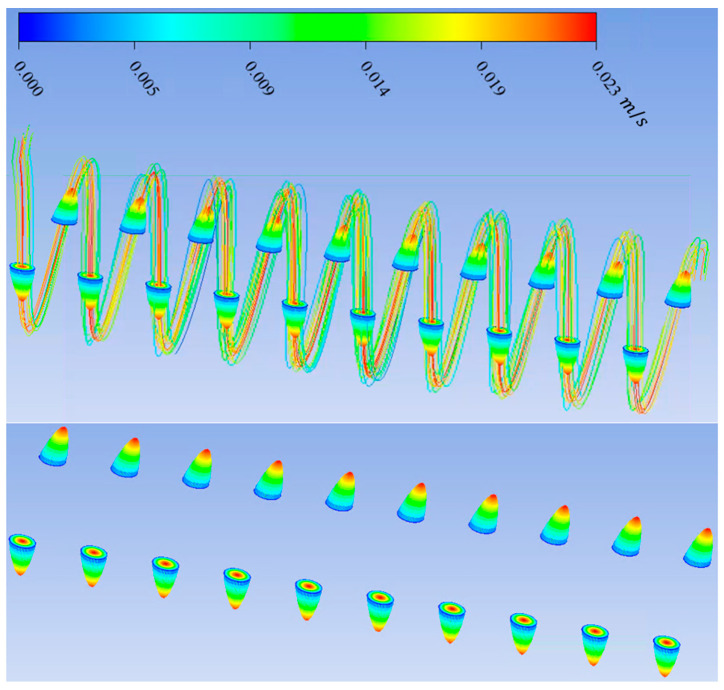
Velocity vectors, streamlines, and velocity contours of a helical connector when $L_{c}=40\;\mathrm{mm}$, $D_{c}=10\;\mathrm{mm}$, d=1.5 mm, and p=4 mm. Working fluid was deionized water, and Re = 400.

In this study, Ansys Fluent was used to solve the Navier–Stokes and continuity equations to predict the streamline, velocity vector, and contour velocity in helical connectors with different geometric characteristics ($L_{c}$, $D_{c}$, d, p) and varying Reynolds numbers. The mesh size was ~0.02 mm, and it was observed that the numerical results were independent of mesh size for all cases. The boundary conditions were inlet velocity and outlet pressure. Inlet velocity was calculated from knowing the volume flow rate and cross-sectional area. The volume flow rate was measured by the syringe pump. Outlet pressure was measured by the pressure sensor at the outlet. The pressure–velocity coupling scheme was coupled. Spatial discretization was, for the gradient, least squares call-based; for pressure, second-order; and for momentum, second-order upwind. The residual absolute criteria for continuity, *x*-velocity, *y*-velocity, and *z*-velocity was 10^−6^. The results of CFD calculation by Ansys Fluent can be seen in Figure 5, Figure 6, Figure 7 and Figure 8. Figure 5 shows the streamlines in a helical connector when the characteristics of the helical connector are $L_{c}=40\;\mathrm{mm}$, $D_{c}=5\;\mathrm{mm}$, d=1.5 mm, and p=4 mm. The working fluid was deionized water, and the Reynolds number was 400. Figure 6 demonstrates the streamlines in a helical connector when the characteristics of the helical connector are $L_{c}=40\;\mathrm{mm}$, $D_{c}=10\;\mathrm{mm}$, d=1.5 mm, and p=4 mm. The working fluid was deionized water, and the Reynolds number was 400. Figure 7 illustrates the streamlines in a helical connector when the characteristics of the helical connector are $L_{c}=60\;\mathrm{mm}$, $D_{c}=10\;\mathrm{mm}$, d=1.5 mm, and p=4 mm. The working fluid was deionized water, and the Reynolds number was 400. Figure 8 shows the streamlines, velocity vectors, and velocity contours inside a helical connector simultaneously when the characteristics of the helical connector are $L_{c}=40\;\mathrm{mm}$, $D_{c}=10\;\mathrm{mm}$, d=1.5 mm, and p=4 mm. The working fluid was deionized water, and the Reynolds number was 400. Figure 5, Figure 6, Figure 7 and Figure 8 demonstrate the impact of the geometric characteristics of each connector on the fluid streamlines when the Reynolds number was 400. Additionally, these figures display the secondary motion for each case. The numerical results indicate that the Reynolds numbers and geometric parameters of the helical connectors have significant impacts on the secondary flow patterns within the connector.

**Figure 9:**
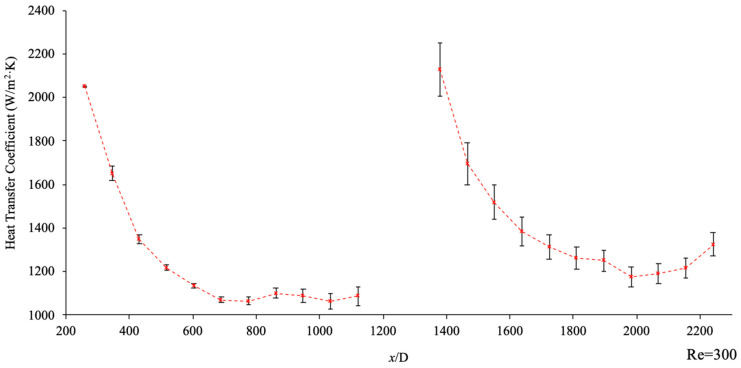
Heat transfer coefficient vs. non-dimensional location, (x/D). Working fluid was deionized water, Re = 300, $L_{c}=40\;\mathrm{mm}$, $D_{c}=10\;\mathrm{mm}$, d=1.5 mm, p=4 mm. The red dotted line displays the variation of heat transfer coefficient in each microchannel and error bars have been included displaying the standard deviation of heat transfer coefficient at each point measured.

An experimental setup was designed to determine the effects the geometric characteristics of helical connectors have on the heat transfer coefficient in the microchannels for different Reynolds numbers. The experimental setup is seen in Figure 1, displaying how the heat transfer coefficient was measured in a two-microchannel system with a helical connector in between. In Figure 9, the variation of the heat transfer coefficient, h, is shown for deionized water at a Reynolds number of 300 as a function of x/D, where x is the instantaneous location measured from the inlet of the two-channel system, and D is the inner diameter of the microchannels. The two curves represent the heat transfer coefficients in microchannel one and two, before and after the connector, respectively, and the heat transfer coefficient standard deviation for each point is represented graphically for clarity. In this study, the experimental data, including microchannel surface temperature for each point, was measured for 1 min, after steady-state conditions were reached. The variation of the heat transfer coefficient for a given point is mostly related to the variation of surface temperature as a function of time for that point. This figure shows that the standard deviation is small compared to the heat transfer coefficient of each point; therefore, the standard deviation was not included in the rest of the figures. Notably, it is seen that standard deviation for the first microchannel is very small compared to that for the second microchannel. This results from the helical connectors’ role in increasing the random motion of molecules in the second microchannel. This increase induces greater variation in the surface temperature along the channel and, thus, the standard deviation is more significant in the second microchannel.

**Figure 10:**
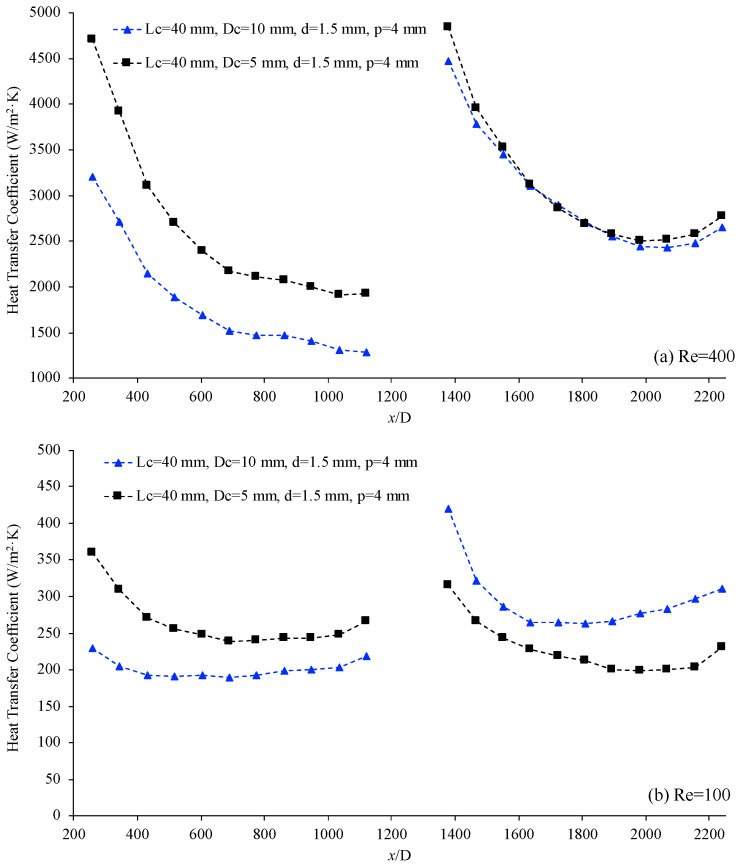
Heat transfer coefficient vs. non-dimensional location, (x/D) for connector (I) (blue) and connector (II) (black). Working fluid was deionized water, (**a**) Re = 400 and (**b**) Re = 100.

Figure 10 below displays the variation of the heat transfer coefficient, h, as a function of non-dimensional location, (*x*/*D*) for two helical connectors with different helical diameters. The characteristics of the helical connectors are (I) $L_{c}=40\;\mathrm{mm}$, $D_{c}=10\;\mathrm{mm}$, d=1.5 mm, p=4 mm and (II) $L_{c}=40\;\mathrm{mm}$, $D_{c}=5\;\mathrm{mm}$, d=1.5 mm, p=4 mm. For both cases, the working fluid is deionized water, and the Reynolds numbers are 400 and 100 for Figure 10a and Figure 10b, respectively. At Re = 400, the heat transfer coefficient is almost same for $D_{c}=10\;\mathrm{mm}$ and 5 mm. However, the level of heat transfer coefficient enhancement for connector (I), $D_{c}=10\;\mathrm{mm}$, is higher than that of connector (II), $D_{c}=5\;\mathrm{mm}$. For lower Reynolds numbers, including Re = 100, connector (I) shows a higher heat transfer coefficient in the second microchannel, demonstrating that in lower Reynolds numbers, the level of heat transfer coefficient enhancement is higher in the connector with the larger helical diameter. For the given conditions, the centrifugal force and secondary flow motion may increase as the diameter of the helical connector decreases. Therefore, the secondary flow motion may act as a stabilizer and decrease the molecular (and possible nanoparticle) random motion. Consequently, for given conditions, the second channel may show heat transfer coefficient reduction as the diameter of the helical connector decreases.

In addition, this figure displays that the heat transfer coefficient is higher in the first channel for connector (II) when Re = 400 (Figure 10a). In this case, while the Reynolds number and working fluid are the same, the level of pressure in the first channel is different, due to the different geometries of the connectors. Practically speaking, different connectors produce different pressure levels in the first channel. The variation in the heat transfer coefficient in the first channel may be related to level of pressure for a given condition when temperature is relatively high. At a high operational temperature, the heat transfer coefficient may adjust, as the level of pressure changes, which might be related to the variation of physical properties of the working fluid, such as enhancement of density and viscosity. Similarly, the effect of the inner diameter of the channel, (d), was investigated, and it was found that connector (I) ($L_{c}=40\;\mathrm{mm}$, $D_{c}=10\;\mathrm{mm}$, d=1.5 mm, p=4 mm) provides higher a heat transfer coefficient compared to that of $L_{c}=40\;\mathrm{mm}$, $D_{c}=10\;\mathrm{mm}$, d=2.5 mm, p=4 mm.

**Figure 11:**
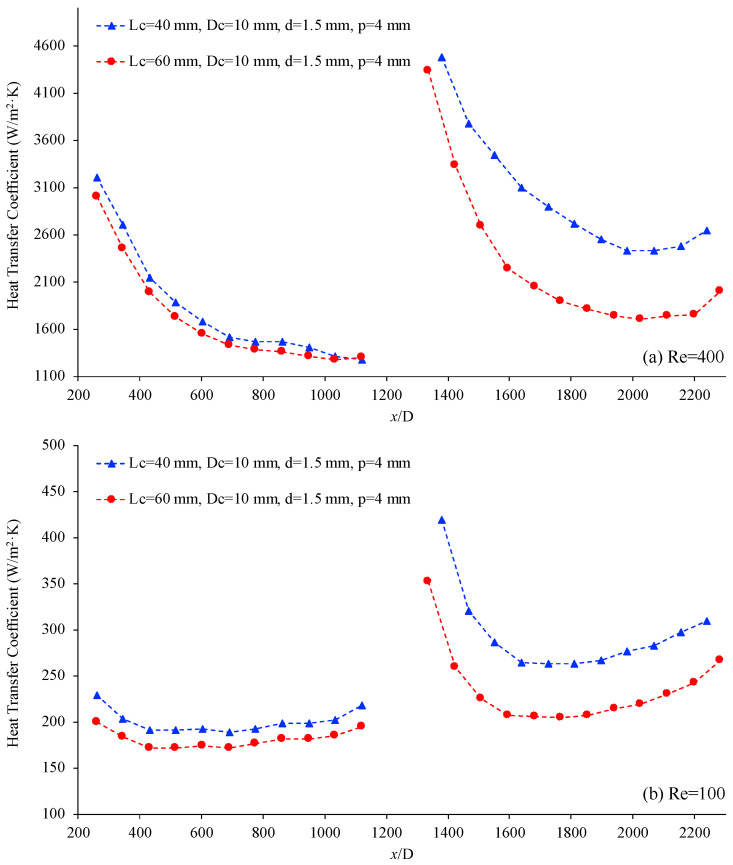
Heat transfer coefficient vs. non-dimensional location (x/D), for connector (I) (blue) and connector (II) (red). Working fluid was deionized water, (**a**) Re = 400 and (**b**) Re = 100.

Figure 11 below shows the variation of the heat transfer coefficient, h, as a function of location (x/D), for two helical connectors with different helical lengths. In this case, the characteristics of the helical connectors are (I) $L_{c}=40\;\mathrm{mm}$, $D_{c}=10\;\mathrm{mm}$, d=1.5 mm, p=4 mm and (III) $L_{c}=60\;\mathrm{mm}$, $D_{c}=10\;\mathrm{mm}$, d=1.5 mm, p=4 mm, the working fluid is deionized water, and the Reynolds numbers are 400 and 100 for Figure 11a and Figure 11b, respectively. This figure demonstrates that the heat transfer coefficient decreases as the length of the connector increases, for the given conditions. As the length of the connector increases, the connector may act more as a stabilizer. If the connector acts like a stabilizer on the fluid flow, molecular random motion may decrease, consequently causing a decrease in the heat transfer coefficient in the second channel as the length of helical connector increases.

**Figure 12:**
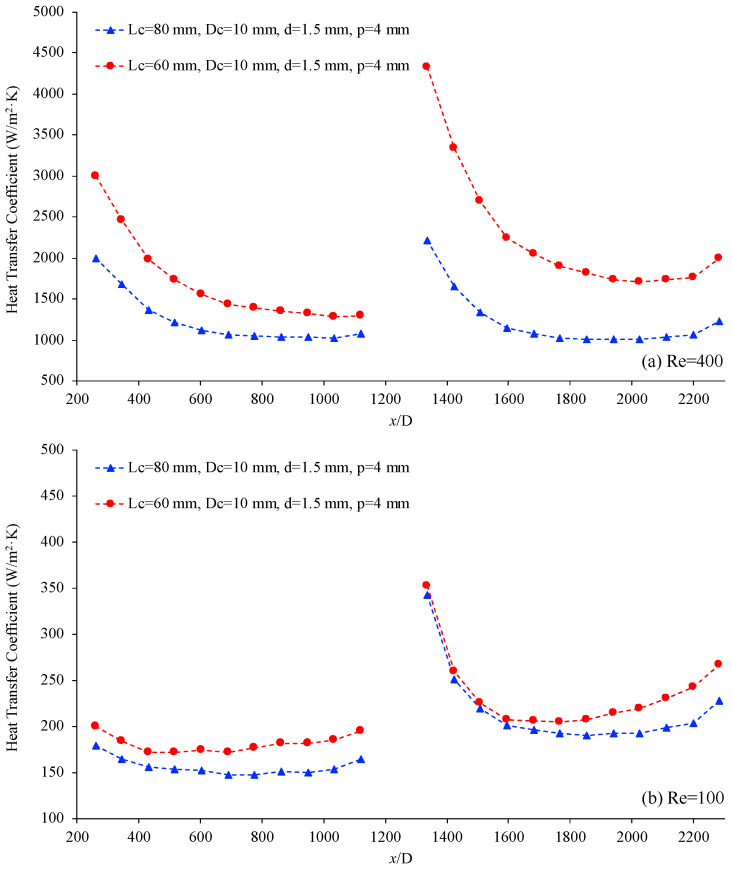
Heat transfer coefficient vs. non-dimensional location, (x/D), for connector (III) (red) and connector (IV) (blue). Working fluid was deionized water, (**a**) Re = 400 and (**b**) Re = 100.

Similarly, Figure 12 shows the variation of the heat transfer coefficient, h, as a function of location, (x/D), for two helical connectors, further demonstrating the effect that helical length has on the heat transfer efficiency of the system. In this case, the characteristics of the helical connectors are (III) $L_{c}=60\;\mathrm{mm}$, $D_{c}=10\;\mathrm{mm}$, d=1.5 mm, p=4 mm and (IV) $L_{c}=80\;\mathrm{mm}$, $D_{c}=10\;\mathrm{mm}$, d=1.5 mm, p=4 mm. The working fluid is again deionized water, and the Reynolds numbers are 400 and 100 for Figure 12a and Figure 12b, respectively. Figure 11 and Figure 12 both demonstrate that as the length of the connector, $L_{c}$, increases, the heat transfer coefficient decreases in the second microchannel. In addition, these figures indicate that connector (I) with $L_{c}=40\;\mathrm{mm}$ is more efficient and results in a higher heat transfer coefficient as the fluid flows through the second microchannel, for the given conditions.

**Figure 13:**
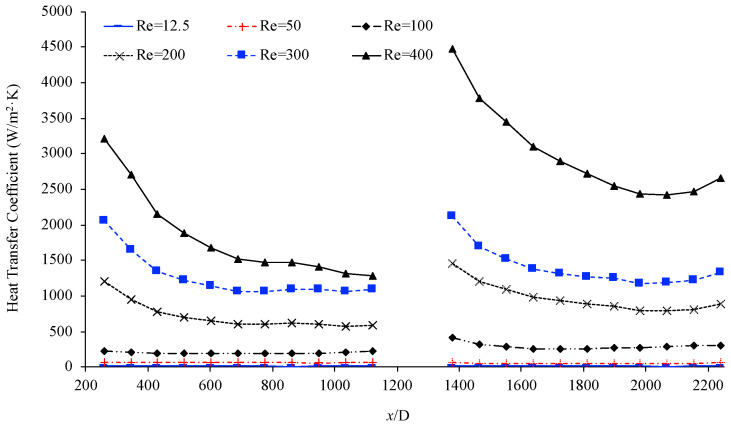
Heat transfer coefficient vs. non-dimensional location, (x/D), with helical connector (I) in between. Working fluid was deionized water, Re = 12.5–500.

Figure 13 below shows the variation of the heat transfer coefficient as a function of non-dimensional location, (x/D), with the use of helical connector (I) in between, at various Reynolds numbers. In this case, the working fluid was deionized water, and the Reynolds number was between 12.5 and 400. This figure shows that the heat transfer coefficient increased as the Reynolds number increased in both microchannels. It has been well established [29,30,31] that as the Reynolds number increases, the molecular random motion of molecules increases, therefore causing an enhancement in the heat transfer coefficient.

**Figure 14:**
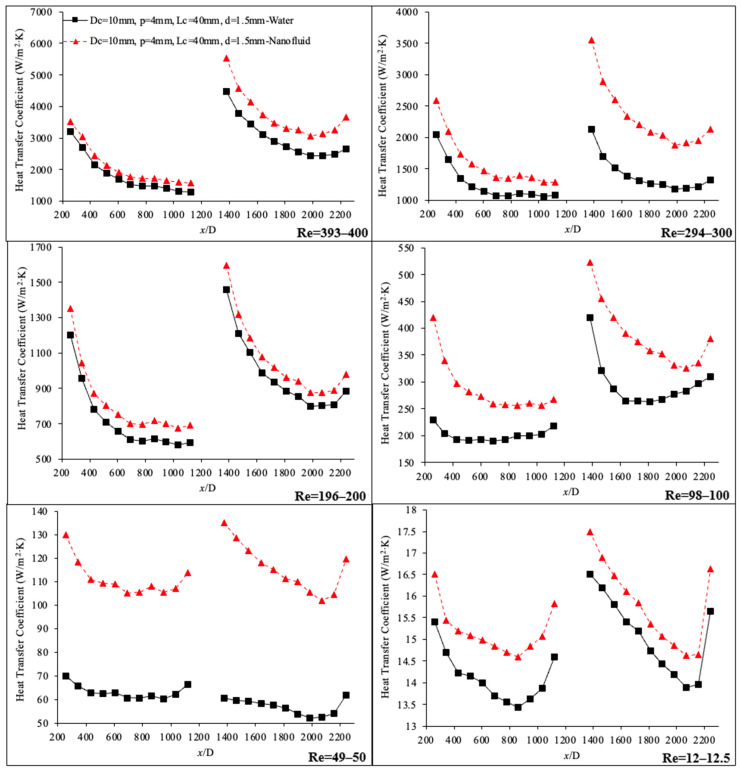
Heat transfer coefficient vs. non-dimensional location, (x/D), in connector (I). Working fluid was deionized water (black) and deionized water–nanodiamond nanofluid (red) with mass concentration of 0.1 wt%, Re = 12.5–400.

Figure 14 shows the variation of the heat transfer coefficient as a function of non-dimensional location, (x/D), with helical connector (I) in between. In this case, the results for deionized water were compared to those of deionized water–nanodiamond nanofluid (mass concentration 0.1 wt%) as the working fluid for Reynolds numbers ranging from 12.5 to 400. In all cases, the introduction of nanodiamond into the base liquid increased the heat transfer coefficient in both channels, which may be attributed to the enhancement of thermal conductivity of the working fluid and increased energy transfer between different layers of flow. The secondary motion also has significant roles on homogenizing the mixture of nanodiamond–water nanofluid. It was found that homogenized nanofluids have higher thermal conductivity [14,15,16]. The increase of energy transfer between layers of fluid in a laminar flow regime may be the result of the induced secondary motion from the helical connector, and this may consequently increase the heat transfer coefficient. Figure 14 also shows that the heat transfer coefficient at the end of the second microchannel tends to increase, with larger enhancements seen at this location for lower Reynolds numbers. This enhancement may be attributed to a reduction in the viscosity of the working fluid due to high fluid temperatures at the end of the second microchannel. The fluid temperature increases as it moves through the microchannel; therefore, fluid viscosity decreases. Additionally, thermal conductivity of the nanofluid and random motion of molecules and possible nanoparticles would also increase with temperature, further enhancing the heat transfer coefficient in the higher temperature region located at the end of the second microchannel. In general, by improving the geometry of the connectors, the stability of the data measurements can be modified.

**Figure 15:**
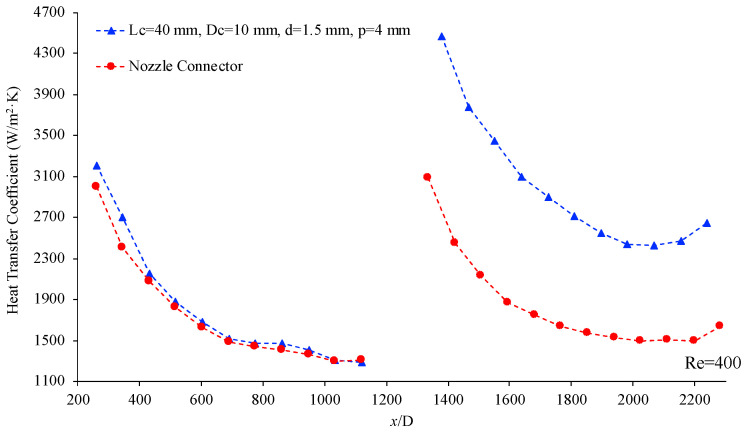
Heat transfer coefficient vs. non-dimensional location, (x/D) for connector (I) (blue) and nozzle-shaped connector (red) [25]. Working fluid was deionized water, Re = 400.4.

Figure 15 below shows the variation of the heat transfer coefficient as a function of location, (x/D), for two different connectors, helical connector (I) and the nozzle-shaped connector shown previously in Figure 3. In both cases, the working fluid was deionized water, and the Reynolds number was 400. This figure displays that as the fluid flows through the connector into the second microchannel, the helical connector can provide greater enhancement in the heat transfer coefficient, suggesting that the helical geometry is superior to that of the nozzle-shaped connector for this application.

## 4. Conclusions

The literature has shown that the superior thermal properties associated with nanofluids can have a significant impact on enhancing heat transfer when the characteristics of nanofluids, such as thermal conductivity, viscosity, temperature, and concentration, are optimized. Additionally, nanoparticles have great potential to transfer energy and momentum between different layers of working fluid if they are distributed homogeneously. It becomes clear that the characteristics of nanofluids must be carefully designed to enhance the heat transfer coefficient for specific conditions, implying that nanofluids should be specifically engineered to meet the demands of the intended application.

The geometry of the system can also have significant impact on its heat transfer capabilities. In general, helical geometry can act as a stabilizer or a mixer on the fluid flow. In this study, it was seen that introducing a helical connector enhances the molecular random motion when it acts as a mixer because of the induced secondary flow motion; therefore, the heat transfer coefficient increases in a microchannel, even at low Reynolds numbers. When the helical connector acts as a stabilizer, the molecular random motion is dampened, resulting in decreased heat transfer capabilities. This study also reveals that under certain experimental conditions, the heat transfer coefficient is positively correlated with the diameter of the helical connector and inversely related to its length. Overall, the influence of the connector on heat transfer efficiency is shown to be dependent on factors such as the Reynolds number and the geometric characteristics of the connector—namely, its length ($L_{c}$), helical diameter ($D_{c}$), (d), inner diameter of the channel, and pitch (p). Optimizing these connector attributes is essential to maximize heat transfer within the microchannel for the given conditions.

The experimental findings indicate that under specified conditions, a connector with the dimensions of $D_{c}$ = 10 mm, d = 1.5 mm, p = 4 mm, and $L_{c}\;$ = 40 mm can significantly enhance the heat transfer coefficient in the second microchannel, particularly when the Reynolds number ranges from 12 to 400. This suggests that such a helical connector design can serve as an effective tool in powerful and miniaturized devices operating in low Reynolds numbers.

## Figures and Tables

**Table 1:** Physical properties of water and water-based diamond nanofluid with concentration of 0.1 wt% at approximately 50 °C [27].

Fluid Property	Water	0.1 wt% Nanodiamond
Dynamic Viscosity (cP)	0.7869	0.8077
Thermal Conductivity (W/m∙K)	0.6381	0.7016

**Table 2:** Helical connector dimensions.

Connector	$L_{c}$ (mm)	$D_{c}$ (mm)	d (mm)	p (mm)
I	40	10	1.5	4
II	40	5	1.5	4
III	60	10	1.5	4
IV	80	10	1.5	4

## Data Availability

Data are contained within the article.

## Nomenclature

**Symbol**	**Meaning (Units)**
A	Cross-sectional area (m^2^)
$c_{p}$	Specific heat (J/kg∙K)
d	Connector inner diameter (m)
$D_{c}$	Helical diameter (m)
D	Microchannel inner diameter (m)
h	Heat transfer coefficient (W/m^2^∙K)
$L_{c}$	Connector length (m)
l	Microchannel length (m)
$\dot{m}$	Mass flow rate (kg/s)
p	Pitch of helix (m)
${\dot{q}}^{\prime }$	Heat flux (W/m^2^)
T	Temperature (°C)
Re	Reynolds number
$\dot{V}$	Volumetric flow rate (m^3^/s)
w	Weight percent
x/D	Nondimensional location along the microchannel
**Subscripts**	**Meaning**
bf	Base fluid
f	Fluid
ND	Nanodiamond
s	Surface
**Greek Symbols**	
ϕ	Particle volume percent
ρ	Density (kg/m^3^)
μ	Dynamic viscosity (N∙s/m^2^)
